# Measurement and clinical usefulness of bilirubin in liver disease

**DOI:** 10.1515/almed-2021-0047

**Published:** 2021-07-09

**Authors:** Armando Raúl Guerra Ruiz, Javier Crespo, Rosa Maria López Martínez, Paula Iruzubieta, Gregori Casals Mercadal, Marta Lalana Garcés, Bernardo Lavin, Manuel Morales Ruiz

**Affiliations:** Service of Clinical Biochemistry, Marqués de Valdecilla University Hospital, Santander, Spain; Commission on Biochemistry of Liver Disease, SEQC^ML^ , Barcelona, Spain; Service of Biochemistry and Molecular Genetics, Hospital Clínic de Barcelona, IDIBAPS, CIBERehd, Barcelona, Spain; Service of Clinical Biochemistry, Hospital of Barbastro, Huesca, Spain; Unit of Liver Disease, Services of Biochemistry and Microbiology, Hospital Universitari Vall d’Hebron, Universitat Autònoma de Barcelona, Barcelona, Spain; Department of Biomedicine, School of Medicine and Health Sciences, Universidad de Barcelona, Barcelona, Spain; Service of Gastroenterology, Marqués de Valdecilla University Hospital, Santander, Spain; Clinical and Translational Research Group on Digestive Diseases, IDIVAL. Santander, Spain

**Keywords:** biomarker, bilirubin, cholestasis, diazo method, liver disease

## Abstract

Elevated plasma bilirubin levels are a frequent clinical finding. It can be secondary to alterations in any stage of its metabolism: (a) excess bilirubin production (i.e., pathologic hemolysis); (b) impaired liver uptake, with elevation of indirect bilirubin; (c) impaired conjugation, prompted by a defect in the UDP-glucuronosyltransferase; and (d) bile clearance defect, with elevation of direct bilirubin secondary to defects in clearance proteins, or inability of the bile to reach the small bowel through bile ducts. A liver lesion of any cause reduces hepatocyte cell number and may impair the uptake of indirect bilirubin from plasma and diminish direct bilirubin transport and clearance through the bile ducts. Various analytical methods are currently available for measuring bilirubin and its metabolites in serum, urine and feces. Serum bilirubin is determined by (1) diazo transfer reaction, currently, the gold-standard; (2) high-performance liquid chromatography (HPLC); (3) oxidative, enzymatic, and chemical methods; (4) direct spectrophotometry; and (5) transcutaneous methods. Although bilirubin is a well-established marker of liver function, it does not always identify a lesion in this organ. Therefore, for accurate diagnosis, alterations in bilirubin concentrations should be assessed in relation to patient anamnesis, the degree of the alteration, and the pattern of concurrent biochemical alterations.

## Introduction

Bilirubin is an orange-yellow pigment of bile that results from the degradation of various heme-containing proteins, especially from hemoglobin catabolism. Heme is broken down into biliverdin, which is converted into unconjugated or indirect bilirubin (UCB). UCB is water-insoluble and enters circulation bound to albumin. In the liver, glucuronic acid is added to UCB (conjugation) to render it water-soluble (direct bilirubin); finally, it is either excreted into bile or recirculated back to the bloodstream, where it is filtrated by the kidneys and excreted through urine [1].

Elevation of plasma bilirubin levels is a frequent finding both in primary [2] and hospital care. All liver lesions induce a decrease in the hepatocyte cell count, which may cause hyperbilirubinemia [3]. Hyperbilirubinemia can originate from an alteration in any stage of bilirubin metabolism: excess production, impaired liver uptake, conjugation defects, or biliary excretion defects [4].

Bilirubin is a well-established marker that is routinely included in biochemical tests for patients with liver dysfunction or any other condition. However, bilirubin is not a sensitive or specific marker of liver function, so a careful interpretation of test results is necessary for accurate diagnosis. Therefore, alterations in bilirubin concentrations should be assessed in relation to patient anamnesis, the extent of the alteration, and the pattern of concurrent biochemical alterations [5], [6]. The purpose of this review is to provide guidance for a correct interpretation of serum bilirubin alterations and ponder its value in differential diagnosis of liver diseases.

## Biochemistry and metabolism

**Figure 1:**
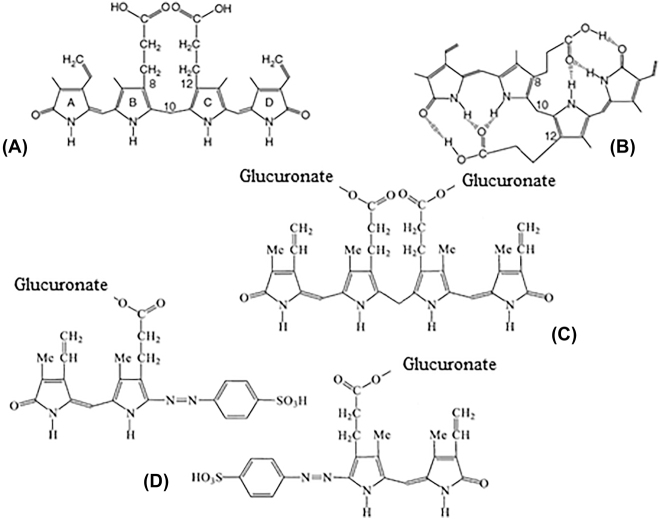
Bilirubin, isomers, and derivatives. (A) Linear or open configuration. (B) Closed configuration, ridge-tiled or mosaic. (C) Bilirubin diglucuronide (conjugated bilirubin). (D) Colored azopyrroles (azopigments) resulting from diazo reaction.

Bilirubin is a tetrapyrrolic product (Figure 1A and B) resulting from the breakdown of the ring of protoporphyrin (hemo). This structure presents various isoforms, of which bilirubin IXα is the major catabolite *in vivo* (near 99%) [1]. Other isoforms such as IIα and XIIIα are found in the bloodstream in a considerably lower proportion, albeit the reference material of bilirubin SRM 916 of the National Institute of Standards and Technology (NIST), no longer available, and other commercial preparations contain significant amounts of these two isoforms [7].

Bilirubin is the product of heme metabolism, especially of hemoglobin resulting from the senescent erythrocytes (80–85%); the remainder fraction comes from inefficient hematopoiesis and other hemo-containing proteins (myoglobin, cytochromes, and peroxidase). The resulting heme, composed of a molecule of protoporphyrin IX and a Fe^2+^ ion, is degraded by the hemo-oxygenase enzyme into a linear molecule of four pyrrolic rings called biliverdin [8]. Free iron (Fe^3+^) and carbon monoxide are also released. Then, biliverdin is converted by the enzyme biliverdin reductase into bilirubin. The major product is the ring-shaped IXα isoform, which is hydrophobic (Figure 1B) [9]. Bilirubin binding to albumin (Kd ≈ 10^−7^–10^−8^ mol/L) prevents isomerization and enables its transportation through the body into the liver [1].

**Figure 2:**
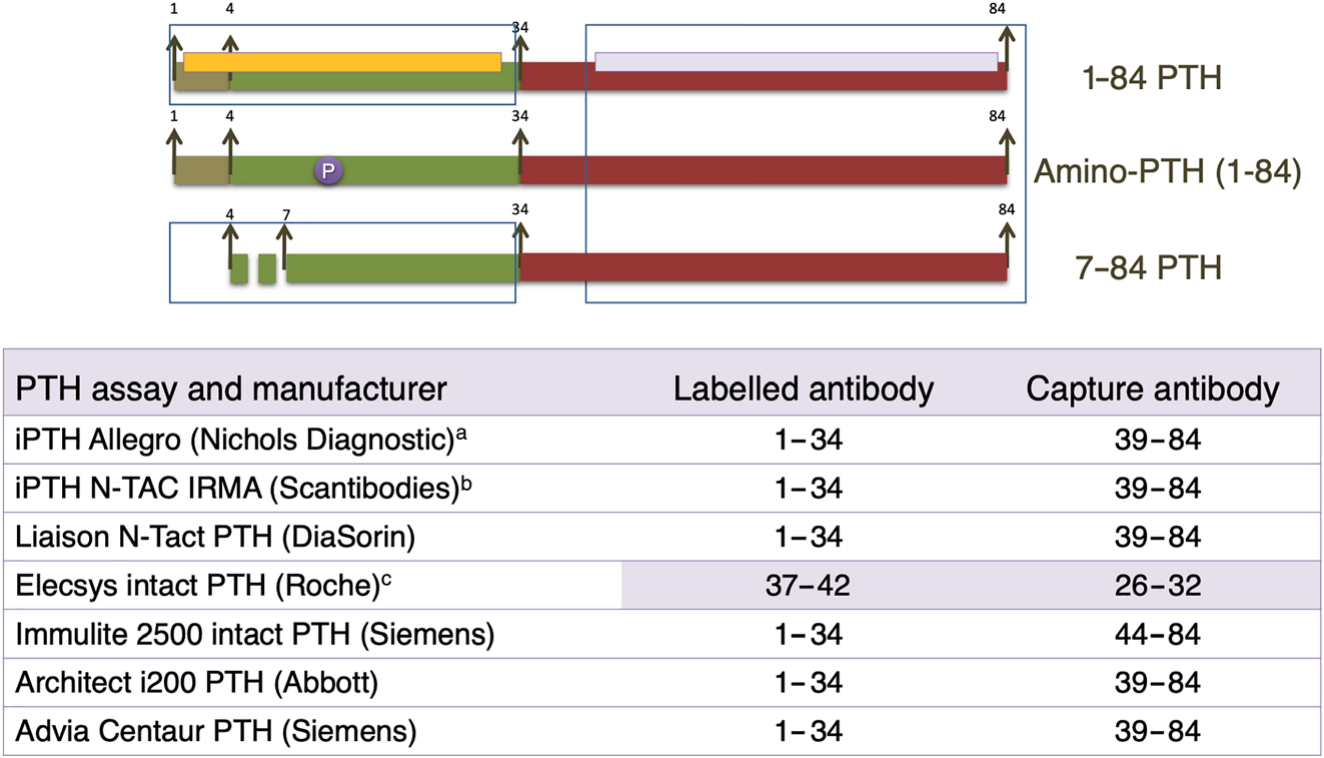
Second-generation assays.Since in the past, they were thought to exclusively measure biologically active 1–84 PTH (7–84 PTH had not yet been identified), second generation assays are incorrectly known as “intact PTH assays”, but they have cross-reactivity with large C-terminal fragments 7–84. As these large fragments, with antagonistic actions to 1–84 PTH, are cleared by the kidneys, their/its percentage increases as glomerular filtration decreases renally. This condition must be taken into account when intact PTH is measured in CKD patients. ^a^This assay, no longer used, was validated by bone histomorphometry and was the assay of reference in most nephrology guidelines. ^b^Isotopic IRMA assay. ^c^Intact PTH assay with a different configuration than the other automated CLIAs. It shows interference with amino-PTH, since its amino-terminal antibody (curiously, the capture antibody) is very distal.

Albumin-bound bilirubin enters the liver through the sinusoids. Organic-anion-transporting polypeptides (OATP) 1B1 and 1B3, encoded in the solute carrier organic anion (SLCO) gene superfamily, mediate bilirubin uptake into the hepatocyte [10] (Figure 2). Once inside liver cells, bilirubin binds water-soluble proteins known as ligandins or Y proteins, which are cytosolic proteins of the glutathione S-transferase family that delay the efflux of internalized bilirubin [11]. Then, in the smooth endoplasmic reticulum, bilirubin is conjugated with glucuronic acid by UDPGT-1A1 to form bilirubin glucuronides [12]. Bilirubin glucuronide returns to cytosol, from which it is transported across the canalicular membrane for excretion into bile, or across the sinusoidal membrane for secretion into plasma, where it undergoes reuptake by the same OATP1B1/3 transporters [6]. In the canalicular membrane, the process is mediated by an ATP-dependent apical transporter, ATP-binding-cassette-C2 (ABCC2), formerly known as MRP2–multidrug related-protein-2 [13].

**Figure 3:**
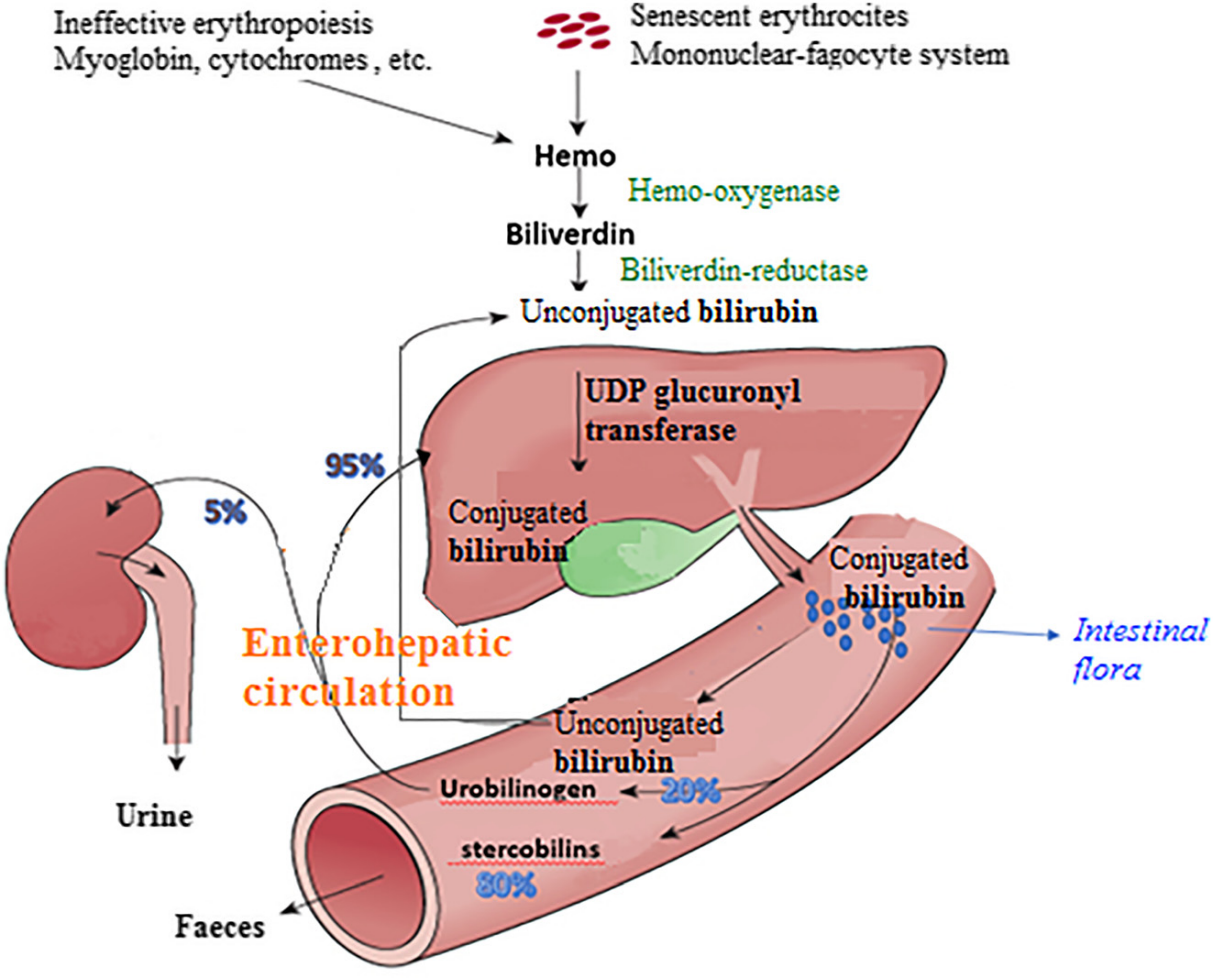
Bilirubin metabolism and recirculation.

Excretion of conjugated bilirubin (CB), which is now polar and water-soluble, is an energy-consuming process. As a result, bilirubin concentration in bile is near 100 times higher than in the cytoplasm of hepatocytes. Water solubility of CB also contributes to prevent reabsorption by the intestine. However, bilirubin monoglucuronides and diglucuronides are relatively unstable compounds that are easily hydrolyzed back into UCB. This effect is facilitated by the action of microbial and intestinal mucosa β-glucuronidase [14]. Bilirubin, converted again into unconjugated bilirubin, is reabsorbed through the intestinal mucosa and returns to enterohepatic circulation (Figure 3). The 25% of bilirubin excreted through the bile duct undergoes this recirculation.

Most of the remainder of UCB in the intestine is reduced by anaerobic gut bacterial flora to form a group of three colorless tetrapyrroles (stercobilinogen, urobilinogen, or mesobilinogen), collectively known as urobilinogens. In the lower gastrointestinal tract, the three urobilinogens spontaneously oxidize to produce the equivalent brownish-yellow bile pigments stercobilinogen, urobilinogen, and mesobilinogen, giving stool its distinctive color. Up to 20% of the urobilinogen produced daily is reabsorbed from the intestine and undergoes enterohepatic recirculation. Most of this urobilinogen is taken up by the liver (via the portal vein) and is reexcreted into bile, whereas a small fraction (2–5%) is excreted into the general circulation and filtered by the kidneys, being detectable in urine.

## Testing methods

Various analytical methods are currently available for measuring bilirubin and its metabolites in serum, urine, and feces. Serum bilirubin is determined by: (i) diazo transfer reaction, currently, the gold-standard measurement method; (ii) high-performance liquid chromatography (HPLC); (iii) oxidative, enzymatic, and chemical methods; (iv) direct spectrophotometry; and (v) transcutaneous methods [15].

Since it was first described by the end of the 19th century, the most widely used methods for measuring serum bilirubin are based on bilirubin reaction with diazotized sulfanilic acid, known as the “diazo reaction” [16]. In 1916, van den Bergh and Müller observed that this reaction was slow in serum of newborns with jaundice and required an accelerator, whereas it was rapid in bile and serum of adults without ethanol addition. Based on this particularity, the terms ‘indirect’ and ‘direct’ bilirubin were coined [17]. The chemical nature of direct and indirect bilirubin was described later in the 1950s, when chromatography enables the identification of three fractions of bilirubin: unconjugated bilirubin (indirect-reaction fraction), bilirubin monoglucuronide, and bilirubin diglucuronide (indirect-reaction fractions) [18]. A fourth fraction results from covalent binding of bilirubin to protein (δ-bilirubin), which is different from the bilirubin–albumin complex in serum [1].

### Diazo method

The reaction of bilirubin with the diazo reagent renders two color azodipyrroles (azopigments) (Figure 1(D)) that can be measured by spectrophotometry, at 530 nm to neutral or acid pH, and at 598 nm to alkaline pH (i.e., by the addition of alkaline tartrate). This reaction is accelerated by alcohol and a variety of other components (i.e., sodium benzoate) causing UCB to dissociate from albumin [19]. In the presence of an ‘accelerator’, conjugated and unconjugated bilirubin are jointly measured (total bilirubin), whereas in the absence of an accelerator, only CB reacts (‘direct bilirubin’).

The difference between Total and CB yields UCB concentration (‘indirect bilirubin’). For the method to be accurate, it is crucial that minimum amounts of UCB react in the direct procedure. The diazo method described by Jendrassik & Grof in 1938 [20] and later modified by Doumas et al. [21] yields total serum bilirubin results which are reproducible and reliable. In this method, the accelerator is a caffeine and sodium benzoate solution. This method has acceptable inter-laboratory transferability and is currently the gold-standard method [15, 21–23]. Its trueness to measure total and direct bilirubin has been assessed by comparing with UCB and bilirubin diglucuronide quantified by nuclear magnetic resonance.

### Chromatography

High performance liquid chromatography has been used to measure total serum bilirubin (TB) after the addition of the four individual fractions or species mentioned above (unconjugated, mono and diglucuronide, and delta-bilirubin). TB values measured by HPLC are consistent with those obtained using the Jendrassik–Grof method [24]. HPLC enabled the identification of the type of bilirubin that endures once initial liver disease has been solved (delta-bilirubin, which has a longer half-life than the other fractions). This technique also helped identifying the nature of the types of bilirubin present in blood or resulting from phototherapy, although this has low or no clinical significance in routine testing [25].

Despite its qualities, HPLC does not have the properties required for being the gold-standard method [15]. That is because HPLC does not measure TB levels with adequate precision and trueness, for a number of reasons: calibration is performed with UCB under the unproven assumption that the other three fractions of bilirubin have the same molar absorptivities as the calibrator; cumulative errors in the measurement of the four species may occur thereby resulting in a considerable total error; and a part of δ-bilirubin can be lost during sample preparation. For routine testing, the method is too complex and insensitive to total bilirubin concentrations <1 mg/dL (17 μmol/L).

### Oxidative methods

Bilirubin may be oxidized by a chemical compound (vanadate) or the enzyme bilirubin oxidase (EC1.3.3.5) to biliverdin, which subsequently oxidizes to initially purple and then colorless products. The concurrent decrease of absorbance at 450–460 nm is proportional to bilirubin concentration. With bilirubin oxidase, total bilirubin is measured at a pH close to 8, whereas direct bilirubin is measured at a pH close to 4. At pH 10, oxidase bilirubin selectively oxidizes CB and a very small amount of UCB, but not δ-bilirubin [26]. The method must be calibrated with UCB in human serum.

### Direct spectrophotometry

This method involves the measurement of absorbance at 437 nm, where bilirubin absorbs at its maximum. Interference from hemoglobin is prevented by testing in a two-component system, measuring hemoglobin at another wavelength and subtracting the correspondent portion. Total bilirubin can also be determined using the cooximeter available in current gasometry systems; in these systems, the spectrophotometer measures the difference between bilirubin and hemoglobin spectra [23].

### Transcutaneous methods

In the 1980s, a range of instruments based on direct spectrophotometry were developed for non-invasive determination of circulating bilirubin. Their performance is acceptable, as compared to the diazo method, with a dispersion of ±2 mg/dL (34.21 mmol/L) [27]. Although transcutaneous measurement cannot replace bilirubin determination in the laboratory, it may be useful for screening. Thus, it provides information at bedside, spares the newborn from the trauma of a puncture, and may reduce the number and cost of serum bilirubin tests. It is also useful to determine whether it is necessary to draw blood from a patient, and is helpful when monitoring phototherapy or exchange transfusion therapies. Several studies and reviews have been conducted to assess the usefulness of transcutaneous measurement of bilirubin in the management of neonatal hyperbilirubinemia [27], [28].

### Urine bilirubin

Only CB is water-soluble and filtered by the kidneys; therefore, its presence in urine indicates conjugated hyperbilirubinemia. The most popular method for testing bilirubin in urine is the reactive strip (elemental), which is impregnated with a diazo reagent (frequently, diazotized dichloroaniline or dichlorobenzenodiazonium fluoroborate). The reactive strip detects bilirubin concentrations as low as 0.5 mg/dL (9 μmol/L); it requires a sample of fresh urine, since bilirubin becomes unstable when exposed to light and stored at room temperature and can oxidize to biliverdin (negative diazo) at the normally acid pH of urine. If testing is delayed, the sample must be kept in a dark place and stored at 2–8 °C for a maximum of 24 h. This method may be affected by positive (ascorbic acid, nitrites) and negative (substances that give urine a brownish/red color such as drugs or metabolites, i.e., rifampicin) interferences [29].

It is worth mentioning that concurrent urobilinogen determination with the same reactive strip provides guidance about the nature of the bilirubin metabolism disorder. Elevated urobilinogen levels with concomitant increased or normal bilirubin are suggestive of increased hemolysis or liver disease, with elevated enterohepatic circulation. In contrast, elevated bilirubin concentrations with normal urobilinogen levels indicate reduced CB secretion into the intestine, as in cases of bile duct obstruction.

### Requirements for bilirubin testing

The requirements for testing bilirubin vary slightly depending on its use. According to CLIA guidelines, a total error of 20% or less is required. Also, the quality standards established by the Interdisciplinary Panel of Experts (CEIEC) of the SEQC/AEFA/AEBM and SEHH establish a variability of 24% as the minimum requirement for testing serum bilirubin [30]. Other objectives, such as those related to biological variability, set 11.3% as the percentage of acceptable error [31]. Although most of the assays and methods currently available meet these standards, it is recommended to verify compliance [5]. Some imprecision persist, especially at elevated bilirubin concentrations [32] and as a function of the matrix used (human or bovine serum) in control and calibration materials [33].

The calibrators available for field methods are based on UCB and CB, with the latter being used to calibrate direct bilirubin measurement methods. The protein matrix of these calibrators is human serum, bovine serum, or a combination of the two. UCB in human serum reacts totally with the gold-standard method and other diazo-based systems available in clinical analyzers. However, its reaction in bovine serum is incomplete and unpredictable. This limitation makes it impossible to determine exact bilirubin values in the calibration material when the protein matrix is bovine serum [34]. According to the literature, ditaurobilirubin in human serum was underestimated by two of the seven clinical analyzers tested, which used calibrators based on bovine serum. Ditaurobilirubin in bovine serum was underestimated by all analyzers and by the method of reference [33]. As a result, calibrators of bilirubin based on a matrix of bovine serum should not be used, since they compromise the accuracy of bilirubin determination tests.

## Clinical significance

Diseases or disorders interfering with bilirubin metabolism may cause an elevation of serum bilirubin concentrations. Elevated bilirubin levels in blood (>1 mg/dL) [35] cause bilirubin deposition in tissues, especially in those containing a large volume of elastic fibers (palate, conjunctiva, among others). Substantial accumulation (generally above 2.5 mg/dL) gives mucosa and skin a yellowish color, which is known as jaundice. Hyperbilirubinemia by itself does not have a poor prognosis [5], since our body has effective detoxification mechanisms (except for neonates). However, it is a sign of impaired bilirubin production or metabolism.

Different approaches have been adopted for the classification of disorders causing hyperbilirubinemia. Based on their location, the disorders inducing hyperbilirubinemia are classified into pre-hepatic, hepatic, or post-hepatic.

### Pre-hepatic hyperbilirubinemia

The term refers to hyperbilirubinemia secondary to excess bilirubin production. The most common cause is accelerated hemolysis. When an elevated bilirubin production rate exceeds the uptake and excretion capacity of the liver, it results in elevated serum UCB concentrations, whereas CB can be normal or slightly elevated. Identifying hemolysis as the cause of hyperbilirubinemia is not challenging, since the patient will exhibit other numerous cueing signs (anemia, elevated reticulocytes, among others) [1]. Since elevated bilirubin is not induced by liver damage, testing will not show alterations in aminotransferases, albumin, or prothrombin activity.

### Hepatic hyperbilirubinemias

The term refers to conditions directly related to liver function. These conditions may affect bilirubin uptake, metabolism, conjugation, and/or excretion, and tests will show elevated CB and/or UCB concentrations.

These diseases are associated with concomitant liver lesions of different severity that may compromise liver function and might follow an acute or chronic course. Especially in the case of acute hepatocellular damage, hepatocellular necrosis induces an increase in transaminases. The hepatocellular diseases that may cause hyperbilirubinemia are: viral hepatitis, alcoholic hepatitis, metabolic steatohepatitis, toxic hepatitis, Wilson disease, hemochromatosis, autoimmune hepatitis, α_1_-antitrypsin deficiency, ischemic hepatitis, and Budd–Chiari syndrome.

#### With concurrent elevation of unconjugated bilirubin

In patients with alterations in liver uptake and/or conjugation, UCB accumulates, and its concentrations increase in blood, whereas CB decreases. This originates a decrease in urobilinogen concentrations, which can be observed in urine and feces (acholia). Bilirubin does not increase in urine (no choluria), since UCB is not water-soluble and is not filtered by the kidneys.

Medications such as rifampicin, chloramphenicol, and probenecid may induce unconjugated hyperbilirubinemia, since it competes with the transporter that carries bilirubin into the hepatocyte.

The elevation of UCB is also associated with inherited disorders affecting conjugation, of which Gilbert syndrome is the most common in adults, affecting 3–10% of the population. UDPGT activity is low. This syndrome does not require follow-up or treatment, since it is a benign disorder. However, it may be a confounding factor when screening for liver disease and is frequently misdiagnosed as chronic hepatitis [3].

When the genetic defect directly affects enzyme production, it causes Crigler–Najjar syndrome (SCN). SCN type 1 is characterized by a total enzyme deficiency that does not improve with induction therapy with phenobarbital. This subtype may be life-threatening due to the neurological toxicity secondary to the deposition of bilirubin in the basal ganglia and nuclei of the brainstem (neonatal kernicterus) [36]. In SCN type 2, enzyme deficiency is partial and responds to phenobarbital and phototherapy, which allows patients reach adulthood. This condition is very rare, with an annual incidence of 1/1,000,000 births [16].

In turn, 60% of newborns and 85% of pre-term infants present with neonatal jaundice. It is generally physiological due to the immaturity of the liver in bilirubin metabolization and excretion, and is limited to the first week of life. If BNC elevation exceeds 5 mg/dL/day, the patient has the risk of developing kernicterus, especially in low-birthweight newborns. This syndrome may be prevented by phototherapy and, in extreme cases, by blood transfusion. Other causes of unconjugated hyperbilirubinemia in neonates may be hemolytic disease, hyperbilirubinemia during the lactation period, and hypothyroidism, among others. The description of these diseases is outside the scope of this study (see references [27] and [36]).

#### With concurrent elevation of conjugated bilirubin

In these cases, bilirubin uptake and conjugation processes work properly, but canalicular excretion is impaired. For this reason, serum CB (as a result of its accumulation in the liver) and UCB are increased [37]. As it occurs in unconjugated hyperbilirubinemia, urobilinogen decreases in urine and feces (acholia) and choluria is detected (bilirubin elevation in urine), since CB is water-soluble and is filtrated by the kidneys.

This elevation can be secondary to inherited diseases compromising bilirubin excretion such as Dubin–Johnson and Rotor syndromes or to intrahepatic cholestatic disorders, viral hepatitis, alcoholic hepatitis, or other liver diseases [11] that include different types of entities:

a) Bile duct disorders: primary biliary cholangitis, primary small-duct sclerosing cholangitis, Caroli’s disease, graft-versus-host disease, adult ductopenia, drugs.
b) Infiltrative disorders: etiology may be infectious (tuberculosis, brucellosis, Q fever, syphilis, leprosy), systemic (sarcoidosis, Wegener’s granulomatosis, lymphoma, amyloidosis), or toxic (allopurinol, sulfa drugs).

Therefore, conjugated hyperbilirubinemia has a high level of specificity for liver damage.

Dubin–Johnson syndrome (SDJ) is a recessive autosomal disorder caused by mutations in the gene encoding ABCC2/MRP2, the protein involved in CB secretion into bile. In clinical terms, it is characterized by chronic, prevailingly conjugated, hyperbilirubinemia without pruritus; from the histopathological point of view, it is characterized by deposits of brownish-black pigments (similar to melanin) in hepatic parenchymal cells [38]. Hepatic enzymes are not altered and, although the level of coproporphyrin excretion does not increase, coproporphyrin I to III ratio reverses. Prognosis is benign.

*Rotor syndrome* is a rare disease of benign hyperbilirubinemia similar to SDJ, although without pigments in the liver. Total coproporphyrins in urine are elevated, of which two-thirds are coproporphyrin I.

Other syndromes causing CB elevation [>1.5 mg/dL (26 μmol/L)] are idiopathic neonatal hepatitis and biliary atresia in neonates. Diagnosis of these entities is challenging. Familial history may be useful for diagnosis of α_1_-antitrypsin, cystic fibrosis, galactosemia, hereditary fructose intolerance, and tyrosinosis.

Other genetic disorders inducing alterations in bile transporters in the canalicular membrane of the hepatocyte are cholestasis of pregnancy, benign recurrent intrahepatic cholestasis, and progressive familial intrahepatic cholestasis. Drugs such as oral contraceptives and cyclosporine may alter CB excretion into the bile canalicular membrane.

### Post-hepatic hyperbilirubinemias

Cholestasis induced by suppression of bile flow prevents partially or totally the release of bile into the duodenum. This suppression of the bile flow is accompanied by the entry of bile into blood. Cholestasis is frequently associated with jaundice, although in some cases there may not be bilirubin retention, which means that cholestasis and hyperbilirubinemia are not equivalent terms. According to the site where bile flow is retained, cholestasis is classified into intra- or extrahepatic cholestasis.

This hyperbilirubinemia is usually accompanied by an increase of cholestasis enzymes (GGT and FA). Fecal material is colorless (acholia) whereas urine has excess color (choluria) and urine urobilinogen is decreased [1]. Extrahepatic cholestasis can be caused by a total or partial physical obstruction of extrahepatic bile ducts. The most common causes include: choledocholithiasis, extrinsic compressions of the bile duct (pancreatic neoplasia, Mirizzi syndrome), disorders of the extrahepatic bile ducts (cholangiocarcinoma, primary or secondary sclerosing cholangitis), and infections (CMV, parasites).

## Bilirubin as a diagnostic and prognostic marker

### In liver disease

As mentioned above, elevation of bilirubin concentrations can be induced by numerous causes and hence, it is a nonspecific marker of liver dysfunction. It is not a *sensitive* marker of liver injury either: a healthy liver can conjugate daily UCB production up to two times without causing an increase in total bilirubin concentrations. Also, the rate of bilirubin excretion is 10 times higher than the rate of bilirubin production [39]. However, hyperbilirubinemia is a long-established marker of liver and bile alterations, and has prognostic value in certain liver diseases [3], [40].

In the hyperacute stage of acute liver failure, bilirubin concentration is relatively low as compared to the substantial elevation of plasma aminotransferase concentrations in plasma. However, in the subacute stage, the situation reverses [41]. In this case, elevated levels of bilirubin in plasma are an indicator of poor prognosis and mortality [42].

Hyperbilirubinemia does not have a prognostic value in patients with acute hepatitis induced by paracetamol, but it does in acute and subacute hepatitis induced by other causes [43]. Bilirubin concentrations >17.6 mg/dL is an indication for hospitalization in patients with acute hepatitis unrelated to the intake of paracetamol [44].

Hepatic cirrhosis can be accompanied by progressive bilirubin elevations. Increased bilirubin concentrations are a relatively late event in chronic liver disease and indicate severe liver dysfunction [4]. In acute chronic liver failure, the elevation of bilirubin favors its dissemination across the blood brain barrier. This situation may be exacerbated by the decrease in albumin concentrations, which impairs bilirubin transport [45]. The consequence is a neurotoxic effect, with progression to a higher level of encephalopathy due to increased concentrations of ammonium ion. High bilirubin concentrations are independent variables associated with the risk of 1-week mortality [46]. Additionally, bilirubin concentrations ≥3.45 mg/dL in patients with chronic liver disease at hospital admission is a predictor of short-term mortality [38].

Cholestatic liver diseases are characterized by bile flow suppression. Advanced disease causes increased bilirubinemia, generally conjugated [16], [43].

It should not be forgotten that elevation of serum bilirubin does not necessarily indicate liver function status. Indeed, the earliest and most accurate marker of liver failure is prothrombin time measured using the international normalized ratio (INR), which should always be included in the evaluation of acute or chronic liver disease.

### In cardiovascular disease

In the 1990s, solid evidence was published of a strong negative correlation between plasma bilirubin concentrations and the risk for coronary artery disease [47], [48], [49]. In the same line, a slight increase in bilirubin concentrations was found to be associated with a lower risk for atherosclerosis in the cohort of the Framingham study [50] and in a cohort of patients with Gilbert syndrome [51]. This suggests that bilirubin is a protective factor against cardiovascular disease independent from standard cardiovascular risk factors.

Recent clinical studies demonstrate that slightly elevated bilirubin concentrations exert protective effects against a variety of oxidative stress-induced diseases, of which atherosclerotic diseases are the most clinically relevant. This issue has been the subject of an excellent review [52].

## Conclusions

Bilirubin is part of the basic study of liver function. There are numerous measurement platforms and methods, being the diazo method the gold-standard. The sample most commonly used is serum or plasma, and also urine, for which optimal pre-analytical conditions are required. Despite its limited sensitivity and specificity, bilirubin is frequently measured for the evaluation of different pathologies related to liver and bile function. Total and conjugated bilirubin concentrations provide guidance about the origin of the alteration. The same occurs with bilirubin and urobilinogen determination in serum and urine. In the hospital context, bilirubin concentrations are very useful for prognosis of acute liver disease and monitoring chronic liver disease. These results must be interpreted in the context of patient anamnesis, degree of alteration, and other clinical laboratory parameters.
